# Initial Experience with ^64^Cu-DOTATATE Digital PET of Patients with Neuroendocrine Neoplasms: Comparison with Analog PET

**DOI:** 10.3390/diagnostics11020350

**Published:** 2021-02-19

**Authors:** Mathias Loft, Camilla B. Johnbeck, Esben A. Carlsen, Helle H. Johannesen, Peter Oturai, Seppo W. Langer, Ulrich Knigge, Andreas Kjaer

**Affiliations:** 1 Department of Clinical Physiology, Nuclear Medicine & PET and Cluster for Molecular Imaging, Department of Biomedical Sciences, Rigshospitalet and University of Copenhagen, DK-2100 Copenhagen, Denmark; mloft@sund.ku.dk (M.L.); camilla.bardram.johnbeck@regionh.dk (C.B.J.); esben.andreas.carlsen.01@regionh.dk (E.A.C.); helle.hjorth.johannesen.01@regionh.dk (H.H.J.); peter.sandor.oturai@regionh.dk (P.O.); 2 ENETS Neuroendocrine Tumor Center of Excellence, Rigshospitalet, DK-2100 Copenhagen, Denmark; seppo.langer@regionh.dk (S.W.L.); Ulrich.Peter.Knigge@regionh.dk (U.K.); 3 Department of Clinical Oncology, Rigshospitalet, DK-2100 Copenhagen, Denmark; 4 Departments of Clinical Endocrinology and Surgical Gastroenterology, Rigshospitalet, DK-2100 Copenhagen, Denmark

**Keywords:** ^64^Cu-DOTATATE, somatostatin receptor imaging, PET/CT, digital PET, solid-state detector, neuroendocrine neoplasms, NEN, NET

## Abstract

The recent introduction of solid-state detectors in clinical positron emission tomography (PET) scanners has significantly improved image quality and spatial resolution and shortened acquisition time compared to conventional analog PET scanners. In an initial evaluation of the performance of our newly acquired Siemens Biograph Vision 600 PET/CT (digital PET/CT) scanner for ^64^Cu-DOTATATE imaging, we compared PET/CT acquisitions from patients with neuroendocrine neoplasms (NENs) grades 1 and 2 and stable disease on CT who were scanned on both our Siemens Biograph 128 mCT PET/CT (analog PET/CT) and digital PET/CT within 6 months as part of their routine clinical management. Five patients fulfilled the criteria and were included in the analysis. The digital PET acquisition time was less than 1/3 of the analog PET acquisition time (digital PET, mean (min:s): 08:20 (range, 07:59–09:45); analog PET, 25:28 (24:39–28:44), *p* < 0.001). All 44 lesions detected on the analog PET with corresponding structural correlates on the CT were also found on the digital PET performed 137 (107–176) days later. Our initial findings suggest that digital ^64^Cu-DOTATATE PET can successfully be performed in patients with NENs using an image acquisition time of only 1/3 of what is used for an analog ^64^Cu-DOTATATE PET.

## 1. Introduction

The role of combined positron emission tomography (PET) and computer tomography (CT) imaging in the diagnosis and follow-up of cancer patients is well established. From the introduction of PET scanners in the 1970s, vast improvements in both PET hardware and software have increased the sensitivity of the modality many fold. Significant improvement in image reconstruction has followed from the inclusion of time-of-flight (TOF) and point-spread-function (PSF) correction, resulting in improved image contrast, spatial resolution and signal-to-noise ratios [1,2,3]. More recently, clinical PET scanners have taken a large leap forward with the introduction of digital solid-state detectors. In a standardized phantom measurement study, the image contrast was >77% better on a digital PET/CT scanner compared with an analog counterpart [4]. The increased detector sensitivity allows for faster image acquisition with a retained signal-to-noise ratio [5]. Alternatively, the increased scanner sensitivity can be traded for a reduced radiopharmaceutical dose, thus reducing the radiation burden on the patients [6].

A digital solid-state detector-based PET scanner was introduced at Rigshospitalet in 2019 with the acquisition of the Siemens Biograph Vision 600 PET/CT (digital PET/CT) scanner. In an initial evaluation of the performance of the scanner, we compared PET/CT acquisitions from patients who underwent a clinical routine PET/CT on both our new digital PET/CT scanner and our Siemens Biograph 128 mCT PET/CT (analog PET/CT) scanner within 6 months. For the comparison, we chose PET/CT acquisitions following the injection of the radiolabeled somatostatin receptor (SSTR)-specific peptide ^64^Cu-DOTATATE. SSTR-PET/CT imaging is routinely used for the diagnosis, treatment planning and follow-up of patients with neuroendocrine neoplasms (NENs) [7,8,9,10,11]. ^64^Cu-DOTATATE was applied first-in-humans at Rigshospitalet and has been thoroughly tested by us for the PET imaging of patients with NENs over the last decade [12,13,14]. Compared with other SSTR-based PET radiopharmaceuticals, ^64^Cu-DOTATATE has the advantage of a flexible acquisition time window ranging from 1 to 3 h after injection with the same lesion detection ability [15]. Recently, we showed that lesion standardized uptake values (SUV_max_) derived from ^64^Cu-DOTATATE PET/CT provided prognostic information on progression-free survival for patients with NENs [16].

In the present study, we focused on patients with NENs grades 1 and 2 (Ki67 proliferation indices <3% and 3–20%, respectively) because this patient group often has relatively stable disease [17] and is frequently investigated with ^64^Cu-DOTATATE PET/CT as part of routine clinical management. This design allowed us to compare the images without introducing additional radiation burden on the patients from additional PET/CTs.

Previous studies have investigated the clinical performance of the Siemens Biograph Vision in terms of the image quality and the lesion detection rates for oncological patients investigated with ^18^F-FDG-PET/CT [18] and the lesion detection rates for recurrent prostate cancer patients investigated with ^68^Ga-PSMA-11 PET/CT [19] and found the digital scanner to outperform its analog counterparts. To our knowledge, no comparison between digital and analog SSTR-PET has been reported to date.

## 2. Materials and Methods

As part of a quality control assessment study at the Department of Clinical Physiology, Nuclear Medicine & PET, Rigshospitalet Copenhagen, we compared ^64^Cu-DOTATATE PET/CT acquisitions from patients with histopathologically confirmed NENs grades 1 and 2 who were scanned on both our Siemens Biograph 128 mCT PET/CT (analog PET/CT) scanner and our new Siemens Biograph Vision 600 PET/CT (digital PET/CT) scanner from November 2018 to August 2019 as part of their routine clinical management. To minimize the potential impact of disease progression and/or regression on the image comparison, we limited the time between the patient’s analog and digital PET/CT acquisitions to 6 months (183 days). Furthermore, we included only PET/CT images from patients with stable disease based on their CTs. We registered NEN-related antiproliferative baseline treatments prior to the analog PET/CT, treatment changes in the interval between the analog and digital PET/CT and treatments following the digital PET/CT. The Rigshospitalet Management approved this retrospective quality control assessment study according to Danish regulations, and the requirement to obtain informed consent was waived.

### 2.1. Image Acquisition and Analysis

The patients received a standard dose of approximately 200 MBq of ^64^Cu-DOTATATE 1 h prior to the PET/CT acquisitions. The analog PET acquisitions were performed on a Siemens Biograph 128 mCT PET/CT scanner with an axial field of view (FOV) of 216 mm and an acquisition time of 4 min per bed position. The analog PET data were reconstructed iteratively using 3-dimensional ordinary Poisson ordered subset expectation maximization (3D-OP-OSEM) with the PSF using the vendor-supplied TrueX algorithm (Siemens Healthineers, Erlangen). Two iterations and 21 subsets were used, including TOF (527 ps), and smoothed by a Gaussian filter (2 mm full width at half maximum). The digital PET acquisitions were acquired on a Siemens Biograph Vision 600 with an axial FOV of 261 mm using continuous-bed motion (FlowMotion^®^) with a bed speed of 1.5 mm/s (equivalent to approximately 90 s/bed position). Reconstruction was similarly performed with 3D-OP-OSEM using the vendor-recommended 4 iteration 5 subset. The PSF and TOF (210 ps) were utilized, and a 2 mm Gaussian filter was applied. Before the PET acquisitions, the patients underwent a diagnostic CT. Unless otherwise contraindicated, the patients received intravenous (IV) iodine-containing contrast prior to the CT. The analog and digital PET/CT acquisitions were analyzed side by side by a team consisting of a nuclear medicine specialist and a radiologist. Lesion-suspicious areas were registered on PET and CT and grouped according to organ or region: bones, intestines, liver, lung, lymph nodes, and pancreas. The number of lesions in each organ or region was capped at 15. Changes in the lesions found on the digital PET/CT compared with the analog PET/CT were assessed on an organ/region basis for each patient. In the case of differences in the number of detectable lesions between the analog PET/CT and the digital PET/CT, patients were followed with relevant imaging data. All the imaging analysis was performed on a SyngoVIA Version VB40A-HF02 (Siemens Healthineers, Erlangen, Germany).

### 2.2. Statistical Analysis

The ^64^Cu-DOTATATE dose, accumulation time (from the radiopharmaceutical injection to the start of PET acquisition), PET acquisition time and time delay between the analog and digital PET/CT are presented as mean and range. Differences in means between the analog and digital PET were compared with paired t-tests. Two-sided *p*-values less than 0.05 were considered statistically significant. All the statistical analyses were performed using the R statistical software (R Foundation for Statistical Computing, Vienna, Austria) version 3.6.1.

## 3. Results

### 3.1. Patients and PET Acquisition Characteristics

Five patients with NENs grades 1 (one patient) and 2 (four patients) had both an analog and a digital PET/CT performed within 6 months with unchanged lesion numbers and sizes on the CT of the analog and digital PET/CT. In all cases, the analog PET/CT was performed prior to the digital PET/CT due to the recent implementation of the digital PET/CT scanner at our institution. The mean (range) time between the PET/CTs was 137 (107–176) days. There were no significant differences between the injected doses (analog PET: 194 (182–209) MBq; digital PET: 192 (182–201) MBq; *p* = 0.8) or radiopharmaceutical accumulation times (analog PET: 75 (62–83) min; digital PET: 69 (57–83) min; *p* = 0.4). The analog PET acquisition time was significantly longer (25:28 (24:39–28:44) min:s) compared to the digital PET acquisition time (08:20 (07:59–09:45) min:s, *p* < 0.001). The imaging parameters for the individual patients are shown in Table 1. The NEN-related antiproliferative baseline treatment and treatment changes in the interval between the analog and digital PET/CT are shown in Table 2. None of the patients had changes in their NEN-related antiproliferative treatment in the interval between the digital PET/CT and the available follow-up imaging.

### 3.2. Image Analysis

**Figure 1:**
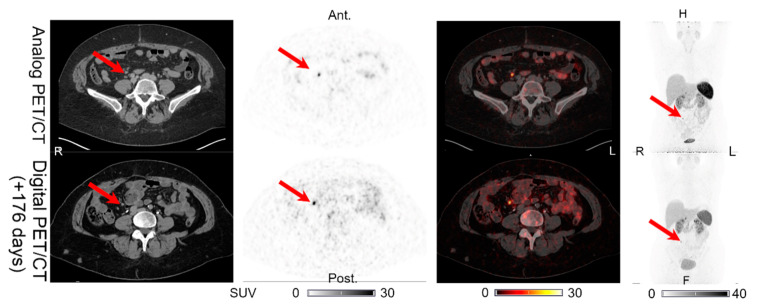
Patient 1: lymph node lesion with structural CT correlates found on both the analog PET and the digital PET performed 176 days later. Arrows on the maximum intensity projection (MIP) and PET/CTs indicate lesion location.

**Figure 2:**
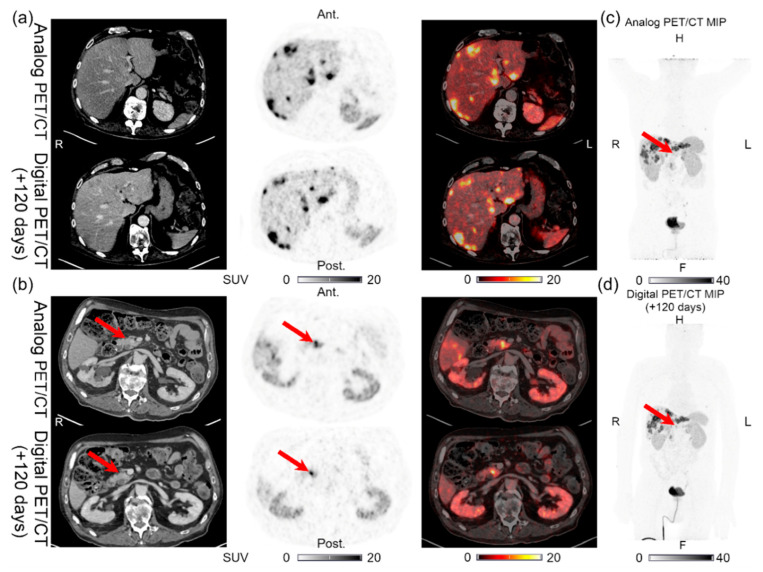
Patient 2: (**a**) multiple liver lesions and (**b**) single pancreatic lesion with structural CT correlates found on both the analog PET and the digital PET performed 120 days later. (**c**) MIP for the analog PET and (**d**) MIP for the digital PET, with arrows showing the pancreatic lesion location in (**b**).

**Figure 3:**
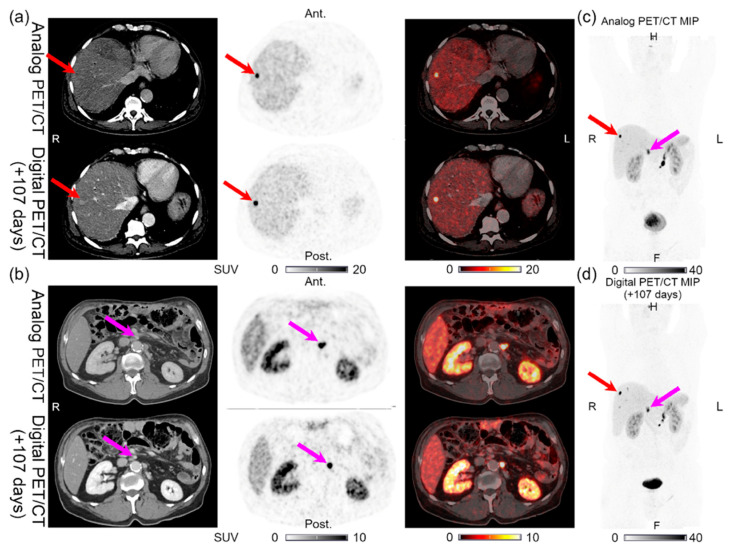
Patient 3: (**a**) liver lesion and (**b**) lymph node lesion with structural CT correlates found on both the analog PET and the digital PET performed 107 days later. (**c**) MIP for the analog PET and (**d**) MIP for the digital PET with arrows showing the lesion location.

**Figure 4:**
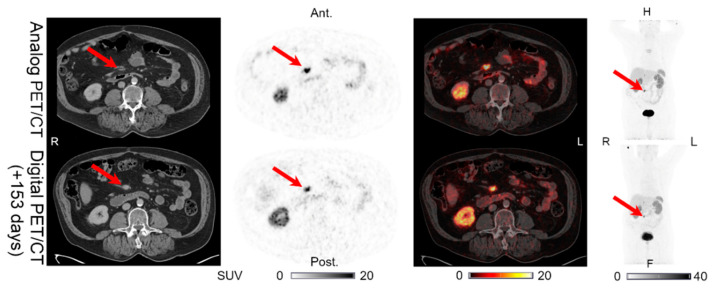
Patient 4: intestinal lesion with structural CT correlates found on both the analog PET and the digital PET performed 153 days later. Arrows indicate lesion location.

**Figure 5:**
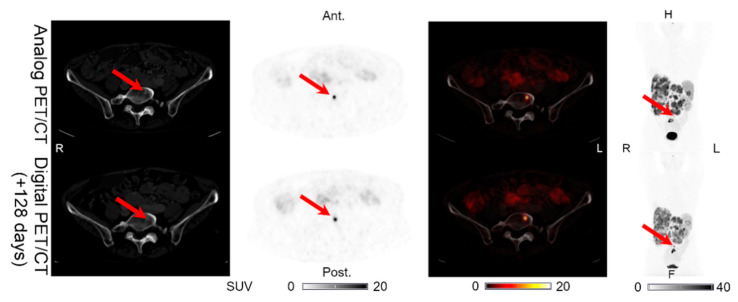
Patient 5: bone lesion with structural CT correlate found on both the analog PET and the digital PET performed 128 days later. Arrows indicate lesion location.

Lesions could be detected on both the analog and digital PET/CT in all the patients with NENs, with the majority of the lesions visible on both PET acquisitions. All 44 lesions detected on the analog PET with corresponding structural correlates on the CT of the analog and digital PET/CT were also found on the digital PET. Representative examples of the lesions found on both the analog PET/CT and digital PET/CT are shown in Figure 1, Figure 2, Figure 3, Figure 4 and Figure 5. All the image findings are summarized in Table 3.

**Figure 6:**
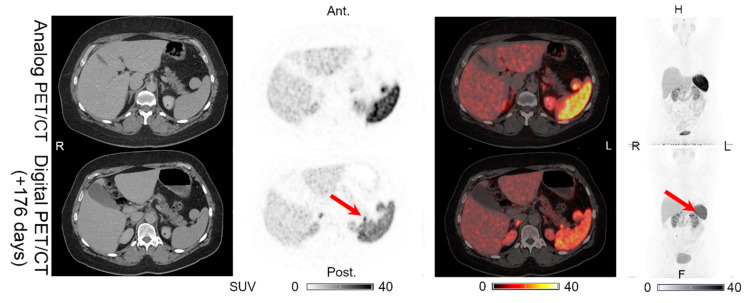
Patient 1: one additional pancreatic lesion found on the digital PET (without CT correlate) performed 176 days after the analog PET/CT, on which the lesion was not detectable. The CT of the digital PET/CT scan was performed without IV contrast. Arrow indicates lesion location.

**Figure 7:**
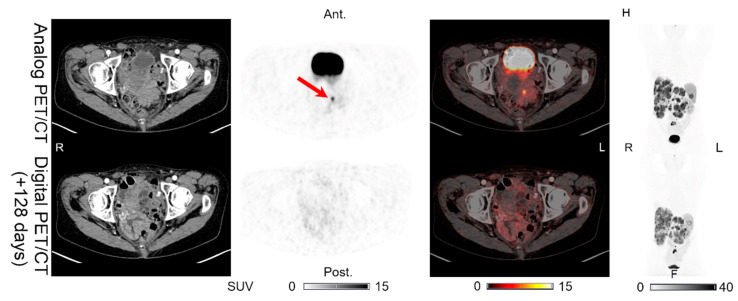
Patient 5: one intestinal lesion (without CT correlate) found on the analog PET but not on the digital PET/CT performed 128 days later. Arrows indicate the locations of the lesions.

In addition to the lesions detectable on both the analog and digital PET/CT, we found differences in the number of lesions detected only on PET in two patients. Patient 1 had one additional pancreatic lesion visible only on the digital PET performed 176 days after the analog PET/CT, on which the lesion was not found (Figure 6). The CT of the digital PET/CT scan was performed without IV contrast. On a CT with IV contrast performed 189 days after the digital PET/CT, the pancreas appeared normal. Patient 5 had an intestinal lesion on the analog PET that had disappeared on the digital PET/CT performed 128 days later (Figure 7). On a follow-up abdominal CT with IV contrast performed 89 days after the digital PET/CT, the intestinal focus could still not be identified.

## 4. Discussion

We compared images from patients with NENs (grades 1 and 2) who underwent both a routine analog and digital ^64^Cu-DOTATATE PET/CT within 6 months. Our main finding was that with the increased detector sensitivity of the digital PET, it is possible to perform a digital ^64^Cu-DOTATATE PET in approximately one third of the time of a conventional analog ^64^Cu-DOTATATE PET (8 vs. 25 min, respectively). All 44 lesions detected on the analog PET, with corresponding unchanged structural correlates on the CT of the analog and digital PET/CT, were found on the digital PET performed 137 (107–176) days later. Using the unchanged CT correlates as surrogates of stable lesions, we believe that the agreement between the analog and digital PET for these lesions supports the use of digital PET with reduced acquisition time. This reduced PET acquisition time adds to the already large flexibility of ^64^Cu-DOTATATE for PET/CT imaging of patients with NENs.

The reduced PET acquisition time minimizes both the risk of movement artefacts and early termination due to patient discomfort. Furthermore, it reduces the effect of bladder filling during the acquisition, which could potentially lead to artefacts in the pelvic area. Another important consequence is that the shortened PET acquisition time could potentially be traded for a “normal” acquisition time (~25 min) with a reduced radiopharmaceutical dose, which would limit the radiation burden on the patient, as suggested by van Sluis et al. [6].

As expected, not all the lesions were detectable on both the digital and analog PET/CT. With the relatively long delay between the PET/CTs, we anticipated some of the patients to show changes in lesions on the later digital PET compared with the analog PET due to the natural course of the disease and/or the effects of treatment. The additional pancreatic lesion in patient 1 was only seen on the digital PET, without a structural correlate on the CT of either the analog or digital PET/CT. However, the CT of the digital PET/CT was performed without IV contrast, and the validity of the finding would, in a clinical setting, be evaluated on follow-up. On the latest available CT with IV contrast, performed 189 days after the digital PET/CT, the pancreas appeared normal. This suggests that the lesion had either regressed following the digital PET/CT or that it represents a false-positive finding on the digital PET. In patient 5, an intestinal lesion was only found on the analog PET, without structural CT correlates. This lesion could represent a true finding on the analog PET that had diminished beyond the detection limit for the later digital PET/CT because of the lanreotide and streptozotocin + 5FU treatment commenced after the analog PET/CT. Alternatively, the focus could be a false finding or a physiological intestinal accumulation, as the normal small and large intestines are known to show variable uptakes of radiolabeled SSTR peptides, which can be mistaken for an intestinal focus [20]. Importantly, the absence of visible correlates on both of the CTs suggests that the absence of the lesions on the digital PET was not a false-negative finding. This is further supported by the follow-up CT imaging that confirmed the absence of intestinal lesions.

Comparisons between analog and digital PET/CT using other radiopharmaceuticals have been reported in the literature. In a prospective study of 20 oncological patients, who successively underwent both digital and analog ^18^FDG PET/CT on Siemens scanners equivalent to the ones used in the current study, van Sluis et al. found additional lesions on the digital PET in 7/20 patients [18]. However, the acquisition time was identical on the analog and digital PET (~180 s/bed position), and the effect of reduced digital PET acquisition time was not explored. In another prospective study, van Sluis et al. compared the effect of simulated reduced acquisition times for digital PET in 30 oncological patients who received a standard dose of 3 MBq/kg of ^18^FDG. They found that with a 3-fold acquisition time reduction (~60 s/bed position), in one of the 30 cases a lesion was missed, leading to change in TNM staging, compared to digital PET using 180 s/bed position [6]. However, no analog PET was performed, making it impossible to assess if the same lesions would have been detected on this system. In a retrospective study of prostate cancer patients, comparing the lesion detection ability in 88 patients imaged with ^68^Ga-PSMA-11 digital PET/CT with matched pairs imaged with analog PET/CT, the digital PET acquisition speed was 0.70 mm/s (~190 s/bed position). Under these conditions, markedly higher numbers of both malignant and benign lesions were found on the digital PET [19].

The study design, with a time delay between the analog and digital ^64^Cu-DOTATATE PET/CT, is a limitation in terms of the direct comparison of the images. Even with a relatively stable disease such as NENs grades 1 and 2, we would expect to see some changes in the patients’ disease despite/because of active treatment. Therefore, we are unable to determine if any newly appearing or missing lesions on the digital PET/CT represent differences in scanner detection ability or are related to the disease and/or treatment. As a consequence, we have refrained from making quantitative comparisons of image quality and radiopharmaceutical accumulation in lesions and organs, as the time delay and potential treatment changes in the interval are confounding factors. For comparison, in the analog vs. digital ^18^FDG PET/CT study discussed above, van Sluis et al. found the SUV_max_, SUV_peak_ and SUV_mean_ in tumors and healthy tissues to be equivalent on the analog and digital PET, while the tumor lesion demarcation, image noise and overall image quality were better on the digital PET [18]. The identical acquisition times for the analog and digital PET limit the generalizability to the current setting, with a reduced digital PET acquisition time. However, the simulation of the reduced PET acquisition time (from 180 to 60 s/bed position) discussed above resulted in unchanged lesion SUV_max_ and SUV_peak_ based on semiautomatic segmentation. On the other hand, the tumor lesion demarcation, image noise and overall image quality were rated lower on the 60 s/bed position PET images [6].

Another limitation of the current study is the small sample size, which limits the generalizability of the findings, although each patient contributed with several lesions, thus increasing the total sample size. However, even with these inherent limitations, we were able to redetect all the lesions on the digital PET with corresponding structural CT correlates. This suggests that the digital PET can successfully be performed in one third the time of an analog PET. Future studies with the inclusion of more patients and shorter time delays between the analog and digital PET/CT are warranted to support these preliminary results.

## 5. Conclusions

We compared images from five patients with NENs (grades 1 and 2) who underwent both an analog and digital ^64^Cu-DOTATATE PET/CT within 6 months. The digital PET acquisition time was less than 1/3 of the analog PET acquisition time (8 vs. 25 min, respectively). All 44 lesions detected on the analog PET with corresponding unchanged structural correlates on the CT were also found on the digital PET performed 137 (107–176) days later. Our initial findings suggest that ^64^Cu-DOTATATE PET can be performed on a digital scanner in 1/3 of the time used for an analog PET. Further studies with the inclusion of more patients are warranted to support these preliminary results.

## Figures and Tables

**Table 1:** Analog and digital PET imaging parameters.

	Analog PET			Digital PET		
Pt	Dose (MBq)	Accumulation Time (min)	Acquisition Time (min:s)	Dose (MBq)	Accumulation Time (min)	Acquisition Time (min:s)
1	182	71	24:39	182	81	07:59
2	198	80	24:39	201	57	09:27
3	209	78	24:39	188	62	08:10
4	189	83	28:44	196	83	09:45
5	190	62	24:39	194	63	06:17
**Mean**	194	75	25:28	192	69	08:20

**Table 2:** Neuroendocrine neoplasm (NEN)-related baseline antiproliferative treatment and changes in treatment.

Pt	Treatment at Analog PET/CT	Changes between Analog and Digital PET/CT
1	None	Lanreotide, 120 mg/4 weeks
2	Octreotide, 40 mg/3 weeks + Interferon-α, 3 MIU/3 weeks	Stopped interferon-α
3	Everolimus, 10 mg/day	No
4	Lanreotide, 120 mg/4 weeks	No
5	None	Lanreotide, 120 mg/4 weeks + streptozotocin/5FU

MIU: million international units.

**Table 3:** Summary of image findings.

Pt	T ^1^	Analog PET/CT ^2^		Digital PET/CT ^2^		Lesion Changes ^3^	Figure	Notes/Follow-Up
		PET	CT	PET	CT			
1	176	LN (1)	LN (1)	LN (1)	LN (1)	UNCH	Figure 1	No IV contrast on CT of digital PET/CT. CT with IV contrast performed 189 days after the digital PET/CT shows normal pancreas.
		PAN (0)	PAN (0)	PAN (1)	PAN (0)	PET (+)/CT (0)	Figure 6	
2	120	HEP (>15)	HEP (>15)	HEP (>15)	HEP (>15)	UNCH	Figure 2a	
		OSS (6)	OSS (2)	OSS (6)	OSS (2)	UNCH	-	
		PAN (1)	PAN (1)	PAN (1)	PAN (1)	UNCH	Figure 2b	
3	107	HEP (5)	HEP (5)	HEP (5)	HEP (5)	UNCH	Figure 3a	
		LN (2)	LN (2)	LN (2)	LN (2)	UNCH	Figure 3b	
4	153	INT (1)	INT (1)	INT (1)	INT (1)	UNCH	Figure 4	Sternal fracture visible on the analog PET/CT.
5	128	HEP (>15)	HEP (>15)	HEP (>15)	HEP (>15)	UNCH	-	CT with IV contrast performed 89 days after the digital PET/CT shows no new intestinal lesions. No other SSTR-PET imaging available.
		INT (1)	INT (0)	INT (0)	INT (0)	PET (÷)/CT (0)	Figure 7	
		LN (1)	LN (1)	LN (1)	LN (1)	UNCH	-	
		OSS (1)	OSS (1)	OSS (1)	OSS (1)	UNCH	Figure 5	

^1^ Time delay in days between the analog PET/CT and the digital PET/CT. ^2^ Summation of the image findings on PET and CT. The number of lesions detected on PET or CT is shown in parentheses for the organs/regions: HEP: liver, INT: intestinal, LN: lymph node, OSS: bone, and PAN: pancreas. ^3^ Lesion changes for each organ/region on the digital PET/CT compared with the analog PET/CT. UNCH: no new lesions or disappearance of lesions on the digital PET/CT compared to the analog PET/CT. In organ/regions with changes in number of lesions, for PET/CT, (0) indicates no changes; (+), new lesions on the digital scan; and (÷), lesion disappearance on the digital scan.

## Data Availability

Data are not publicly available due to protection of personal data and medical confidentiality.
